# Reconfigurable optical logic in silicon platform

**DOI:** 10.1038/s41598-024-56463-x

**Published:** 2024-03-11

**Authors:** M. A. Ruhul Fatin, Dusan Gostimirovic, Winnie N. Ye

**Affiliations:** Department of Electronics, Carleton University, 1125 Colonel By Drive, Ottawa, ON K1S 5B6 Canada

**Keywords:** Integrated optics, Optoelectronic devices and components, Integrated optics, Optoelectronic devices and components

## Abstract

In this paper, we present a novel, scalable, and reconfigurable optical switch that performs multiple computational logic functions simultaneously. The free-carrier depletion effect is used to perform non-volatile switching operations due to its high speed and low power consumption. We adopt the concept of optical memory using a phase-change material to realize the non-volatile reconfigurability without a constant power supply, in addition to providing a large operating bandwidth required for reconfigurability. The proposed reconfigurable optical logic architecture is realized in a compact microdisk resonator configuration, utilizing both the carrier-depletion-based modulation and phase-change optical memory. This is the first time these two modulation schemes are implemented in the same optical microdisk for the purpose of reconfigurable optical logic.

## Introduction

Current Central Processing Unit (CPU) architectures can execute processing and memory operations simultaneously and can operate at ultrafast speeds for real-time applications such as video analysis, detection of fast-moving objects and LIDAR analysis^1–3^. However, recent trends in transistor size provides evidence that the Moore’s law is reaching its saturation^4^. Additionally, the limitation in architecture, known as the von Neuman bottleneck^5^ where the system’s performance is often limited by the speed of accessing memory, becomes a severe challenge in emerging memory-intensive applications. In particular, photonic systems offering broad bandwidth and high speeds allow for a shift to a completely different computing paradigm in developing systems such as Photonic Neural Networks (PNN)^6,7^, Photonic Quantum Information^8^ and Optical Field Programmable Gate Arrays (FPGA)^9,10^. In a large-scale integrated (LSI) optical logic architecture—an optical equivalent of FPGA, an essential building block is an optical logic gate with reconfigurable switching modes.

The use of thermo-optic (TO) or electro-optic (EO) effects to actively tune silicon photonic devices is well known. But EO effects have a refractive index tuning range in the order of only 0.0001 and TO effects are slow with a refractive index tuning range in the order of 0.01. Thus, a large phase shift usually requires long waveguides, inevitably increasing the footprint and complexity of the device. Resonator structures can solve the issue of footprint but at the cost of the bandwidth of operation. Furthermore, these switching mechanisms are volatile and require a constant power supply.

The most widely used mechanism to perform switching operations in silicon photonic platforms is the free carrier depletion (FCD) effects due to their high speed and low power consumption^11–13^. For FCD based microresonators speed of 128 Gb/s has been achieved (with PAM4 modulation) with an electro-optic bandwidth of 50 GHz^14^. But FCD effects are volatile and not inherently reconfigurable. Devices based on optical phase change (O-PCM) materials have been studied because of their large index change properties and reconfigurability^15^. The phase change introduced by O-PCM materials can be transitioned from the amorphous to the crystalline state upon electrical or optical excitation and is a non-volatile operation. Recent research has also shown that, some O-PCM materials such as $\mathrm {Ge_2Sb_2Te_5}$ (GST) can be tuned quasi-continuously and the intermediate states between the amorphous and crystalline states can be conveniently achieved at room temperature^16^. However, the electro-thermal switching of PCM materials is relatively slow, of the order of 500–1000 Kb/s^17^. It would be advantageous to combine both switching mechanisms in our proposed reconfigurable logic architecture.

In this paper, we present a novel optical switch that can be applied to an optical directed logic architecture that is compact in size, scalable and reconfigurable. Our proposed switching employs both carrier depletion and phase-change material-based switching on the same device. Optical logic cells and architectures with hybrid electro-optic plasmonic switches have been presented in^18^. This design includes a plasmonic waveguide metal as an integrated heater to switch the states of the PCM. The switching architectures in this work employ only the non-volatile switching of the PCM to realize logic operations. In our proposed reconfigurable switch, the FCD effects are utilized to perform the switching controlled by the logic signal; while the PCM is used to switch between the operation modes (e.g., OR and AND logic operations) of the logic cell. The PCM switching, while slower than the FCD effects, provides a wide tuning range and is appropriate for signal reconfiguration. On the other hand, the high speed and low power FCD switching performs the logic operations with a high bit rate. Since we are using a non-volatile switching on the operation modes, they can be reconfigurable without exerting any constant power source. Our proposed device can perform multiple logic operations at a single wavelength, reducing the complexity and footprint of the device. This approach requires fewer resonator structures than previous demonstrations^12,13,19^, saving device footprint and power consumption, and improving scalability.

## Results and discussion

### GSST cladded disk resonator

Using either an optical or electrical (through Joule heating) excitation, an Optical Phase Change Material (O-PCM) can be reversibly transitioned from a low-loss amorphous state to a high-loss and absorptive crystalline state. In this work, we propose the use of a relatively new class of, Ge-Sb-Se-Te (GSST), which is derived from the conventional Ge-Sb-Te (GST) by partially substituting Te with Se. Compared with the commonly used O-PCM such as GST and $\mathrm {VO_2}$, GSST has lower optical loss in both its amorphous and crystalline states at the telecommunication bandwidths of interest. One may use the emerging $\mathrm {Sb_2Se_3}$ instead, as it has advantages over GSST such as lower absorption losses, lower switching temperature, and potentially easier integration onto standard SOI integrated photonic platforms^20^; however, its slower crystallization speeds in the millisecond range^21^ and dependence on film quality limit^22^ its suitability for high-speed applications compared to the more mature GST-based systems. For the phase change activation, optical heating by applying laser pulses provides a fast switching speed (switching speed can be on the order of sub-ns) with efficient energy consumption. But this method is not feasible for large-scale integration since the task of aligning laser pulses on each of the O-PCM samples can be slow, diffraction-limited, and inaccurate. O-PCMs can also be switched by evanescent coupling of optical pulses to O-PCMs, but would introduce complexity in light routing. In our model, we choose to integrate external indium tin oxide (ITO) heaters on the top of the O-PCM. ITO has low optical loss and can serve as a viable option for heating material with good thermal conductivity.

**Figure 1:**
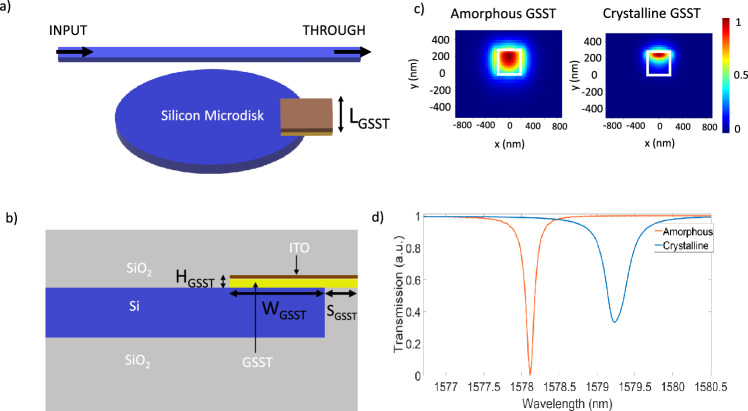
(**a**) Schematic of GSST on Silicon Microdisk with ITO heater on top. (**b**) Cross-section of the right half of the disk. (**c**) Evolution of a quasi-TE mode traversing along a Si waveguide with GSST on top. (**d**) Demonstration of redshift of resonance wavelength with changing the GSST from amorphous to crystalline. The orange and blue plot indicates response in amorphous and crystalline states respectively. Lumerical’s FDTD simulation were performed on a Silicon microdisk of 5 μm radius and 0.22 μm height. Other parameters are: $\mathrm {L_{GSST}}$ = 1.5 μm, $\mathrm {W_{GSST}}$ = 0.4 μm, $\mathrm {S_{GSST}}$ = 0.6 μm, $\mathrm {H_{GSST}}$ = 0.025 μm, height of ITO layer is 0.01 μm. The amount of redshift we see is around 1.5 nm.

We consider a microdisk resonator where it is partially covered by a GSST cladding element, as shown in Fig. 1a. Thin films of GSST can be simply deposited through a thermal evaporation process from a bulk material. The detailed process steps can be found in^23^.

Light entering through the input port gets partially coupled to the whispering gallery mode inside the microdisk. Using the simple Fabry-Perot theory we can calculate the resonant modes^24^:

1 $$\begin{aligned} \begin{aligned} 2\pi R_{disk} n_{eff} = m. \lambda _{m} \end{aligned} \end{aligned}$$

**Figure 2:**
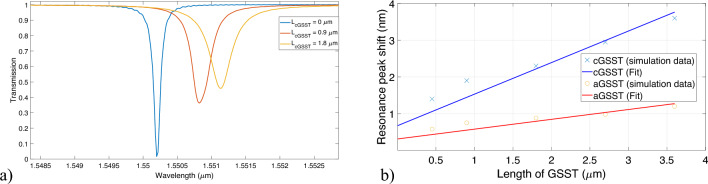
(**a**) Transmission spectra is redshifted as we gradually increase $\mathrm {L_{cGSST}}$. (**b**) The shift of the resonance peak with gradual increase in the $\mathrm {L_{cGSST}}$ or $\mathrm {L_{aGSST}}$.

Equation (1) provides the resonant condition (at wavelength $\lambda _m$) for the ring resonator of radius $R_{disk}$ where $n_{eff}$ is the effective refractive index of the whispering gallery mode, and *m* is the mode order. Here we use a small patch of 400 nm $\times$ 1.5 μm GSST on our 5 mm-radius microdisk. The exact size of the GSST is not critical, as long as it covers a part of the whispering gallery mode in the disk. Figure 1b shows the cross-section of the microdisk. In Fig. 1c, we present the modal field distribution of a quasi-TE mode through a typical Si waveguide (450 nm × 220 nm) with a GSST overcladding. When the GSST is in its amorphous state, the mode is more confined within the Si waveguide core because GSST has an effective waveguide refractive index of 2.80281 + i0.000111. When the GSST is in its crystalline state the mode is more absorbed towards the GSST cladding with a higher waveguide refractive index of 2.8057 + i0.00057. In Fig. 1d we can see a redshift of the resonance peak after the crystallization of the GSST cladding. The dimensions of the microdisk simulations are provided in the figure description. Note that apart from $\mathrm {L_{GSST}}$ the rest of the parameters are kept constant in the remainder of the paper. We can then optimize the length of the GSST to design the device tailored to our needs.

Figure 2a shows the evolution of transmission spectra as the length of GSST strip is increased. It can be observed that the shifts in resonance peak follow a linear trend (shown in Fig. 2b). After fitting the results, we see that the resonant shift per unit GSST length ($\Delta \lambda / L$) for amorphous and crystalline GSST are 0.2674 nm/μm and 0.8596 nm/μm respectively, at 1550 nm. Thus, we can express the spectral shift quantitively as^25^:

2 $$\begin{aligned} \frac{\Delta \lambda }{L_{GSST}} \approx \frac{\Delta n_{eff} \lambda _0}{2 \pi R_{DISK} n_{g}} \end{aligned}$$

    Where $n_{g}$ is the group index of the whispering gallery mode (with no GSST overlay) of the microdisk and $\Delta n_{eff}$ is the change in the real part of the effective index. Using our simulation parameters, we find $\Delta / L$ to be 0.203 nm/μm and 0.73 nm/μm for amorphous and crystalline GSST respectively, which is in close agreement with our fitted values presented in Fig. 2b. Equation (2) can also be used to find the critical length of the GSST cladding with given switching conditions. For example, to achieve a shift of 130 pm in wavelength a critical length of approximately 430 nm of GSST is needed. Thus, this relation offers basic guidelines for the geometrical dimensions of the GSST in the design.

In optimizing the GSST length for this design, two primary factors demand consideration. Firstly, achieving a balanced Extinction Ratio (ER) between the amorphous and crystalline states is crucial, ensuring a relatively high ER in both states. Additionally, as detailed in subsequent sections, the wavelength shift for changing the states needs to match the wavelength shift caused by the carrier depletion phenomenon. The fitting parameters in Fig. 2b were eventually used to obtain a ballpark value of the required GSST length.

### Electrothermal switching of O-PCM with ITO heater

In this section, we describe the details of our 3D time-dependent simulations performed to model the heat transfer of GSST material. Table 1 shows the simulation parameters we used in the Multiphysics simulations in COMSOL.

**Table 1:** Parameters used for thermal simulations in COMOSOL multiphysics.

Material	Thermal conductivity (W/m K)	Heat capacity (MJ/$\mathrm {m^3}$/K)	Electrical conductivity(S/m)
ITO Heater (10 nm)	1.16^26^	2.6^26^	$8.6\times 10^3$^27^
GSST (amor)	0.18^28^	1.5^28^	0.2^17^
GSST (crys)	0.43^28^	1.9^28^	5000^17^

**Figure 3:**
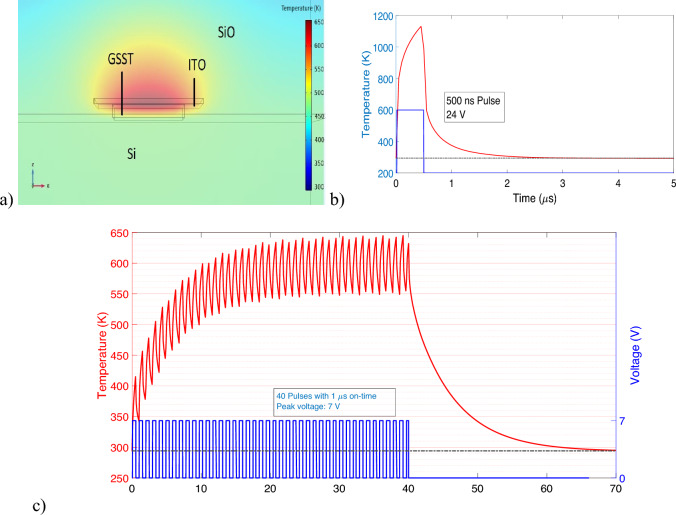
Electro-thermal switching from amorphous to crystalline through Joule heating and vice versa. (**a**) Heat map produced by Joule heating. (**b**) Temperature average in the GSST layer as a function of time for rectangular pulse heater. For amorphization, the temperature is kept above 900 K (amorphization temperature) by applying a single rectangular pulse of 24 V. (**c**) For crystallization, the average temperature is kept above 523 K (crystallization temperature) but below 900 K for more than 20 μs.

We use a rectangular ITO heater of dimensions $3\times 3$ μm^2^ and height 10 nm to heat the GSST layer. Figure 3a shows the heat map produced by Joule heating the GSST material. Figure 3b and c show the switching dynamics of amorphous to crystalline and vice versa. Around 900 K of temperature is required to melt the GSST into amorphous state, whereas for inducing re-crystallization, the GSST needs to be heated above the crystallization temperature ($\sim$523 K) but below the melting point ($\sim$900 K), for a critical amount of time of 20 μs^28^. It can be seen from Fig. 3b that a 500 ns pulse of 24 V is required to melt the crystalline GSST and transition it to its amorphous state. From Fig. 3c, at around 20 μs the average temperature of the GSST reaches above 523 K; while 56 pulses with 700 ns on-time with a peak voltage of 15 V are required to maintain a relatively stable temperature for an additional 20 μs. Through simulations, it can be seen that the crystallization to amorphization process for the GSST can be performed with a cycle duration of 1.5 μs. The reverse process of amorphization to crystallization takes a cycle length of approximately 80 μs. Electro-thermal analysis of a similar structure was performed in^29^. Their simulation showed a 24 V pulse of $\sim$2.5 μs duration is required to switch the GSST strip from crystalline to amorphous state and a 5 V pulse train of $\sim$80 μs duration is sufficient to induce re-crystallization. Utilizing optimized heater designs, like positioning tungsten in direct contact within a lateral configuration alongside the GSST film, enables the attainment of fast transition times-approximately 1 μs for crystalline to amorphous transitions and 20 μs for amorphous to crystalline shifts^28^. Pt nanostrips can also be a good candidate to efficiently conduct heat to the GSST film with short-duration current pulses and with peak power estimated to be below 10 mW^30^.

### Carrier depletion in silicon microdisks

**Figure 4:**
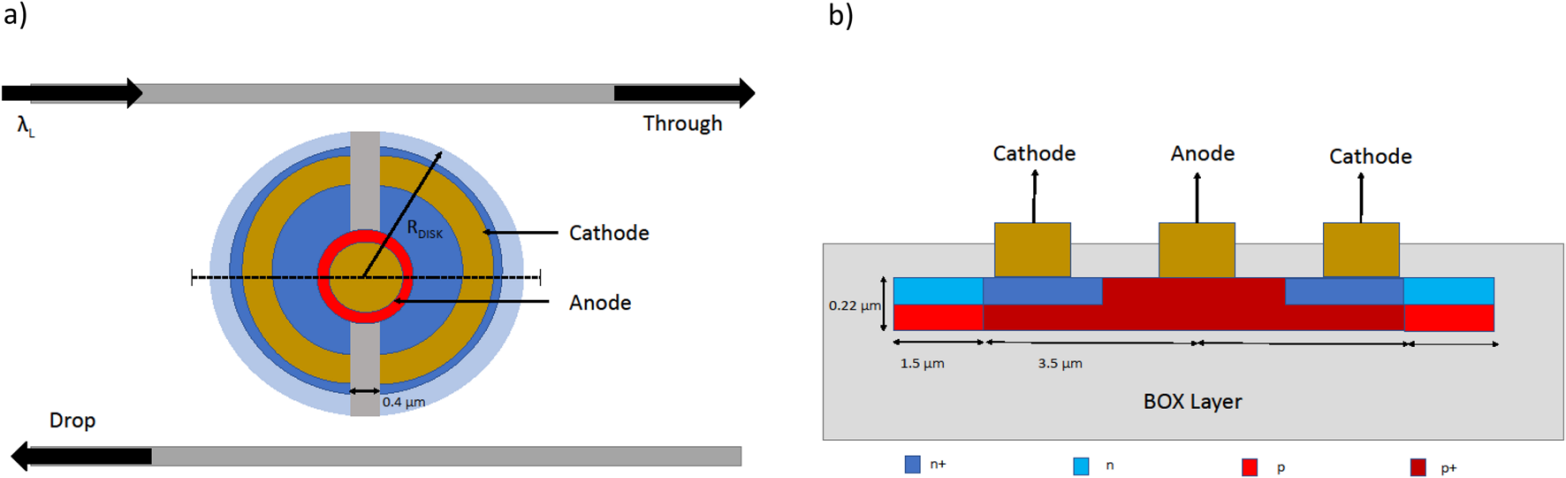
(**a**) Top view of the microdisk with double-shift vertical pn junction. The gold and graded region describe the metal anode and cathode, the red and blue describe p-type and n-type Silicon respectively. The darker red and blue indicate p+ and n+ type Silicon respectively. (**b**) Cross section of the microdisk along the dashed line in (**a**). The microdisk is buried in oxide with the p,n,p+ and n+ regions defined.

**Figure 5:**
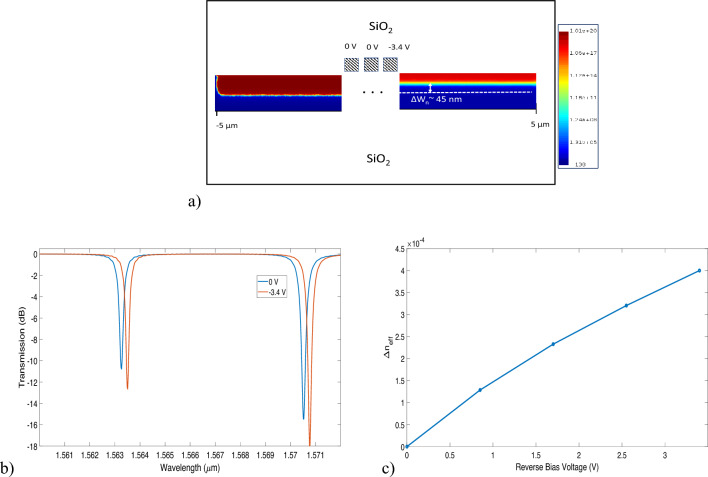
(**a**) Cross section view across the diameter of the microdisk. The color map shows the simulated carrier profile of free electrons of the vertical junction when 3.4 V of reverse bias is applied on the right of the microdisk. The right side of the disk shows a 45 nm of larger depletion width. (**b**) Transmission response at the drop port of the microdisk with applied biases of 0 V and − 3.4 V. (**c**) Refractive index change of the vertical junction with applied reverse voltage.

In this work, we use a double-shift vertical pn junction microdisk resonator as shown in Fig. 4a. The vertical pn junction is more desired than lateral junctions in rings because the depletion region developed in vertical junctions is more distributed along the whispering gallery mode and thus reduces the voltage requirement^19^. Figure 4a presents a schematic of pn junction controlled microdisk modulator. We perform finite element method (FEM) simulations using Lumerical’s DEVICE suite (Version 2020 R2.0) to calculate the charge density profile that would eventually be imported into the 2D MODE model (Ansys Lumerical’s finite difference eigenmode solver) to simulate the refractive index change due to applied bias. The refractive index values are then used to create a new material model for the FDTD and varFDTD simulations. For the purpose of implementing logic operations, we choose a Si disk of radius 5 $\mathrm {\mu m}$ with an oxide cladding. We believe this device size is optimum in having both the carrier depletion and PCM switching in the same microdisk. The bus waveguides are 0.4 $\mathrm {\mu m}$ wide and the spacing between the disk and waveguides is 0.16 $\mathrm {\mu m}$. Figure 4b shows a cross-section of the vertical junction with defined concentration regions. We use n-doped Silicon of concentration of $4\times 10^{18} \; \text{cm}^{-3}$ and p-doped Silicon of concentration $2\times 10^{18} \; \text{cm}^{-3}$ at the edge of the Silicon disk. Underneath the anode, it lies the highly doped p+ Silicon of concentration $1\times 10^{20} \; \text{cm}^{-3}$; while below the cathodes it contains the highly doped n+ Silicon of concentration $2\times 10^{20} \; \text{cm}^{-3}$. Figure 5a shows the depletion region for the vertical junction with an applied bias of − 3.4 V. The transmission response of the disks is simulated using the varFDTD approach in Lumerical. The varFDTD approach effectively approximates a 3D geometry into a 2D geometry by converting the vertical waveguide structures into effective dispersive materials. Thus, this approach provides reasonable accuracy with speed comparable to that of 2D FDTD simulations. The applied bias shifts the resonance peak by 123.75 pm/V (i.e., 396 pm for a − 3.4 V applied bias). The disk has a low Q factor of approximately 6284 with FWHM = 251.2 pm and a transmission coefficient of 0.987. In Fig. 5b the transmission response of the disk resonator in the drop port is presented with applied biases of 0 V and − 3.4 V. Figure 5c shows the change in refractive index of the vertical junction with reverse bias voltage between 0 V to 3.4 V. The total switching delay is defined as $\tau _{switch} = \tau _{RC} + \tau _{photon}$, where $\tau _{RC}$ is the RC constant of the metallic contacts and $\tau _{photon}$ is the photon lifetime^31^. The 3 dB bandwidth of the optical, electrical, and overall operation can be described as,

3 $$\begin{aligned} \Delta f_{3dB,optical} = \sqrt{\sqrt{2} - 1} \frac{c_0 \delta \lambda }{\lambda _0^2} \end{aligned}$$

4 $$\begin{aligned} \Delta f_{3dB,electrical} = \frac{1}{2\pi R C} \end{aligned}$$

5 $$\begin{aligned} \Delta f_{3dB,optical} = \frac{\Delta f_{3dB,electrical} \Delta f_{3dB,overall}}{\sqrt{(\Delta f_{3dB,optical})^2 +(\Delta f_{3dB,electrical})^2}} \end{aligned}$$

where, $c_0$ and $\lambda _0$ indicate the vacuum speed of light and resonance wavelength respectively. $\delta \lambda$ indicates the linewidth, *R* and *C* are the resistance and capacitance of the double vertical junction. The pn junction has a capacitance of 8.48 fF with switching energy of 21.7 fJ/bit at − 3.4 V bias. The overall NRZ bit rate of the device is calculated to be 27.74 Gbps. The switching energy per bit, albeit higher than other reported designs^13,19,31^, is negligible compared to the energy on the order of micro-Jules, required by the electrothermal switching of the amorphous to crystalline states. For this reason, the FCD effect is unlikely to induce any phase change operation in the PCM.

### Switching and logic architecture

**Figure 6:**
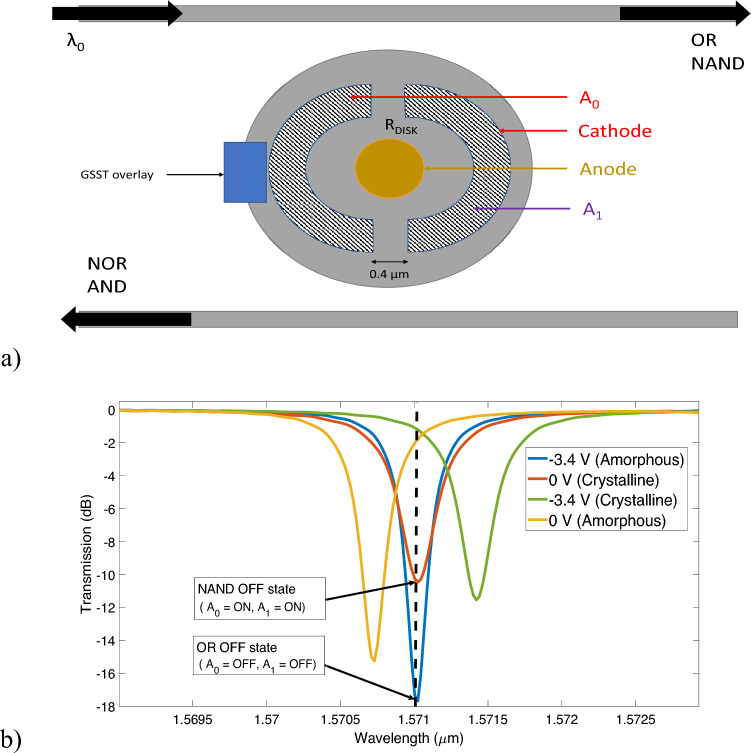
(**a**) Top of the microdisk with a PCM strip. The PCM strip has a length of 400 nm, a width of 200 nm and 25 nm of height. (**b**) Transmission response under different states of PCM and pn-junction. Two logic operations can be carried out at wavelength 1.571 μm.

In a reconfigurable switch, two types of resonance tuning are used. One is used for logic operation; while the other is for sending a reconfiguration signal to switch the mode of the operation. For switching the logic signal we use a disk resonator with a vertical pn junction as shown in Fig. 6a. The blue rectangular strip is the phase change material GSST that can be tuned to switch between two logic operations such as NAND to OR and vice versa. We can give some consideration to how this particular design can be fabricated. The modulator is made first as mentioned in^19^. It is a mostly reasonable process where a pn junction is added to an SOI waveguide with a specific pattern that is unique to our vertical junction design. Subsequently, the PCM is deposited atop this structure^17,23^, as illustrated in Fig. 6a, while appropriately arranging metal contacts for both the junctions and the PCM.

A continuous-wave (CW) light is sent to the waveguide at a fixed wavelength. When the disk is on resonance most of the input light is collected at the drop port, with a little output in the through port. A photodetector at the end of the through and drop port can perform the O/E conversion needed to analyze the logic output. The signals applied to the contacts $A_0$ and $A_1$ of the microdisk can be considered as inputs to the logic operation and the photodetector responses as the output of the operation.

**Table 2:** Logic table for NAND and OR operations.

Logic mode	$A_0$	$A_1$	Output (through port)	Loss (dB)	ER (dB)
NAND (crystalline)	0 (− 3.4 V)	0 (− 3.4 V)	1	− 1.21	17.7
	0 (− 3.4 V)	1 (0 V)	1	− 1.21	
	1 (0 V)	0 (− 3.4 V)	1	− 1.21	
	1 (0 V)	1 (0 V)	0	− 18.91	
OR (amorphous)	1 (− 3.4 V)	1 (− 3.4 V)	1	− 3.08	10.5
	0 (0 V)	1 (− 3.4 V)	1	− 3.08	
	1 (− 3.4 V)	0 (0 V)	1	− 3.08	
	0 (0 V)	0 (0 V)	0	− 13.6	

For the proof of concept, we consider that the crystalline state of the PCM will perform NAND/AND operations and the amorphous state will perform OR/NOR operations. In the crystalline state, we consider that having a 0 V and − 3.4 V bias between the contacts indicates logical ‘1’ and logical ‘0’ states, respectively. Table 2 shows the logic table for the NAND operation when the PCM is in a crystalline form. Table 2 shows that applying a 3.4 V reverse bias voltage (which is logical ‘1’ for crystalline state) will shift the resonance peak and most light will be collected at the photodetector in the through port and set the output of the NAND operation to high (logical ‘1’). Under the same conditions, the photodetector at the drop port will be performing the AND operation. Now, let us explore the OR operation using the same device. We can send a reconfiguration signal to the PCM to change its state to amorphous. The logical ‘1’ and ‘0’ states represent a bias of − 3.4 V and 0 V respectively for the amorphous state as shown in Table 2. Figure 6b shows the transmission response at the through port of the device under different switching states of the PCM and pn-junction. Figure 6b illustrates an ideal situation where the resonance peak of the amorphous state with a − 3.4 V bias and a peak of the crystalline state with a 0 V bias occurs at the same operating wavelength of 1.571 μm. With 0 V of bias on the contacts most of the light will go to the drop port, setting the OR output to ‘0’; and with a − 3.4 V bias at any of the contacts, most light will go to the through port setting the OR output to ‘1’. Thus, we can now perform OR and NOR operations at the through and drop ports, respectively, by switching the $A_0$ and $A_1$ contacts. As per requirements of the switching architecture, the blueshift of the resonance peak due to switching the PCM state from crystalline to amorphous needs to be approximately equal to the redshift of the resonance peak due to − 3.4 V of applied bias. Keeping this requirement in mind, Eq. (2) was used to obtain an approximate value of the length of the PCM strip, and small sweeps of its width and position were performed in Lumerical to finalize the dimensions of the strip. In real applications, microresonators would need thermal tuning to stabilize the spectrum. It is possible to achieve partial crystallization/amorphization by fine-tuning the amount of heating^3,32^. Consequently, the PCM can serve for effective thermal calibration.

### N-x logic operation

**Figure 7:**
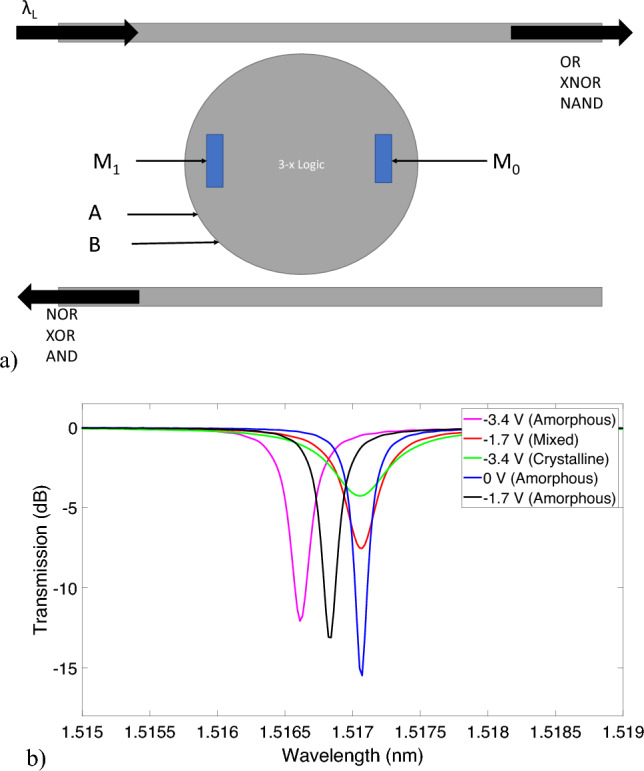
(**a**) Schematic of a logic cell with 3x operations in a single disk and at a fixed wavelength. This cell can be scaled up to nx operations. (**b**) Transmission response of a logic cell of similar functionality. The blue, red and green curves represent amorphous state with 0 V bias, mixed state with − 1.7 V bias and crystalline state with − 3.4 V bias respectively. The black and purple curves represent amorphous states with − 1.7 V and − 3.4 V applied Bias. We can see that by changing the state of the PCM we can shift the resonance peak and align them with the initial state. This can be utilized for our 3x operation logic cell.

It is possible to achieve n-x operations of logic in a single microdisk by having more than one PCM strips on a single disk. But it will be at the expense of higher optical loss since the PCM is highly absorptive. In this section, we demonstrate 3x logic operations in a single microdisk with a vertical pn-junction and overlayed with two PCM strips. Figure 7a shows the schematic of the logic cell.

Following a similar strategy as in the previous section, we consider that when both the PCM strips are set to amorphous, the logic unit will perform the NAND/AND operations; and when both set to crystalline, the OR/NOR operations can be obtained. When one PCM strip is set to crystalline and the other to amorphous (which is referred to as the “mixed state”) the disk will perform the XNOR/XOR operation. Table 3 shows the states of the PCM and pn-junction bias applied to demonstrate these logic operations. In this table, we are only showing the scenarios, where the microdisk is on resonance and most light goes to the drop port. For all other cases, the disk will be off-resonance and the light goes to the through port.

**Table 3:** Blueprint for 3x logic operation.

Logic operation	PN junction labels	PCM state	ER (dB)	Optical loss (dB)
NAND	A = B = 0 V (‘1’), OUTPUT = ‘0’	$M_0$ = $M_1$ = amorphous	15.5	− 1.59
XNOR	A or B = − 1.7 V, OUTPUT = ‘0’	$M_0$ = amorphous, $M_1$ = crystalline	7.54	− 4.72
OR	A and B = − 3.4 V (‘0’), OUTPUT = ‘0’	$M_0$ = $M_1$ = crystalline	4.3	− 8.16

From the transmission responses of the through port in Fig. 7b we observe that the disk is on resonance at 1.571 nm (for cases where OUTPUT = OFF for all 3 logic operations). Thus, we only require a single wavelength source to perform all 3 logic operations. The biggest caveat in this design is the low transmission and low ER for OR operation. Since we are using two PCM strips in this design, the whispering gallery mode experiences a higher loss when both of them are under the highly absorptive crystalline state. Microring modulators with U-shaped PN-junctions have been demonstrated in^33,34^ with improved modulation efficiency and lower loss. With better modulation efficiency and low-loss resonator structures, we can use similar principles as in this section to obtain n-x logic operations on the same micro-resonator structure. As an example, GSST clad bistable 2 × 2 crossbar switches can be explored for similar applications^30^.

This concept of employing multi-functional microresonators for logic operations was previously investigated in^35^. Unlike our proposed device, these multifunctional devices require fine-tuning the input signal’s wavelength. In contrast, our device seamlessly switches between various functionalities utilizing PCMs. Additionally, the PCMs serve the dual purpose of stabilizing signals against environmental changes, eliminating the requirement for external heaters to maintain stability. However, the referenced design holds an advantage over ours in terms of lower transmission loss. Nevertheless, leveraging PCMs for reconfiguration signals allows our device to maintain operational modes without a constant power supply, thus improving the overall power consumption.

### N-bit adder

**Figure 8:**
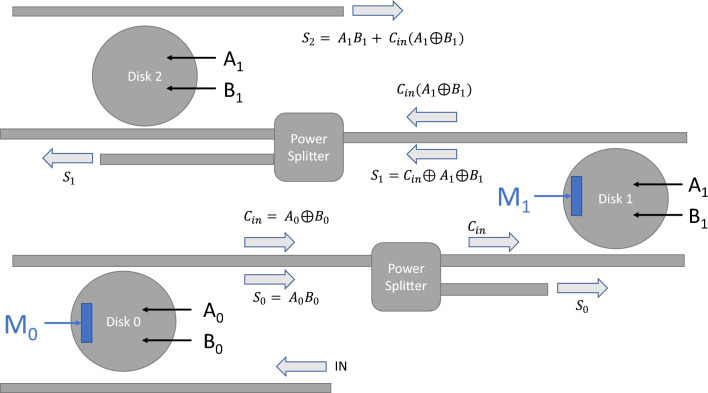
Schematic of a 2-bit optical adder that is using 3 microdisks. Disk 0 and Disk 1 have both PCM and pn-junction control while Disk 2 has only the pn-junction control.

Figure 8 shows the schematic of a 2-bit adder with Disk 0 and Disk 1 being set up in a similar configuration as in Fig. 6a. Disk 2 can be operated with just pn-junction control. The equations governing the full adder can be written as

6 $$\begin{aligned} S_1 & = A_1 \oplus B_1 \oplus C_{in} \\ S_2 & = C_{out} = A_1.B_1 + C_{in}(A_1 \oplus B_1) \end{aligned}$$

**Table 4:** for Optical Adder operation with PCM strips.

$M_0$ state	$M_1$ state	Output collected	‘1’ Threshold (a.u.)	‘0’ Threshold (a.u.)
AND	N/A	$S_0$	0.56	0.014
XOR	XOR	$S_1$	0.4	0.018
XOR	AND	$S_2$	0.29	0.021

**Figure 9:**
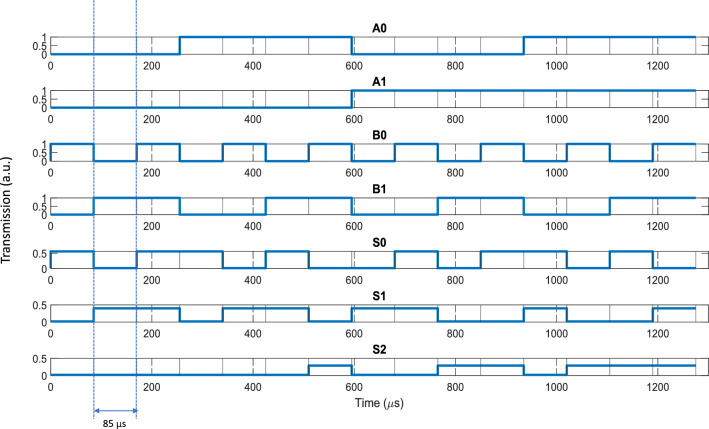
Waveform of a full adder with ERs given in Table 2.

With PCM strips $M_0$ and $M_1$, we can control whether Disk 0 or Disk 1 performs AND or XOR operation. Let us assign the crystalline state to the AND operation and the amorphous state to the XOR operation. When Disk 0 is configured to AND operation, it is expected to produce $S_0 = A_0 B_0$ and the power splitter can be used to collect $S_0$. With Disk 0 set to XOR, it produces the CARRY output $C_{in} = A_0 \oplus B_0$ which is taken as the input to Disk 1. Again, with $M_1$ being set to XOR we can collect $S_1$ through the power splitter. Finally, the output of Disk 1 is collected with $M_1$ set to AND which is eventually used as the input to Disk 2. Disk 2 then performs the OR operation to collect $S_2$ at its output whose expression is defined in Eq. (6). Table 4 shows the states of the PCM strips used to collect the adder outputs. The table also shows the threshold values of outputs ‘1’ and ‘0’ estimated from the ER values given in Table 2. In Table 4 it is evident that to collect all three outputs, the states of $M_0$ and $M_1$ are switched from crystalline to amorphous and vice versa for each bit operation. Hence, we estimate that each bit operation would require a maximum time of approximately 85 μs. Figure 9 shows the estimated waveforms of the three outputs ($S_0$, $S_1$, and $S_2$) with all logic combinations of the four input signals ($A_0$, $A_1$, $B_0$, and $B_1$). The four input signals themselves are switched with much faster carrier depletion effects (of the order of pico-seconds) and their time delays were ignored in the figure. A full optical adder of such architecture improves upon the number of resonant structures used in^19^. Optical adders like this can be a key component of an Electronic-Photonic arithmetic logic unit (EPALU) that can allow high-speed computing with lower power consumption^36^. It should be noted that PCMs generally have reliability issues with limited writing times. The aging of the amorphous state in phase-change memory (PCM) and the resultant resistance drift phenomenon pose some challenges within this technology especially for multi-level storage devices^37^. Utilizing two-level storage in our proposed logic architectures provides a relatively simpler approach to adjusting the impacts of aging compared to the challenges posed by multi-level storage configurations. The threshold levels of the output signals can be periodically calibrated over time to counteract the impact of aging.

## Conclusion

We present an optical logic cell with a compact microdisk resonator configuration where switching is achieved through the free carrier dispersion effects and the state change of a phase change material. In comparison to other designs, this proposed logic cell can perform multiple logic operations at a single wavelength, which allows for the construction of a complex logic architecture without the need of multiple wavelength sources or wavelength-division multiplexing. The use of phase change material to reconfigure the logic unit promotes a more energy-efficient alternative as the switching mechanism does not require a constant power supply. Using Multiphysics modeling we estimate that it is possible to achieve reconfigurable switching with the highest switch cycle of 80 μs. Meanwhile, from the drift-diffusion calculations, we estimate that the vertical pn junction can provide around an NRZ switching speed of 27 Gbps. This novel method of integrating two switching mechanisms on a single microdisk enables a compact, fast switching and energy efficient logic unit that can eventually be a building block of a reconfigurable directed logic architecture. We also demonstrate that performing operations on binary numbers using silicon photonic optical logic is more efficient when multiple signals are processed at once, using n-bit operations. This saves time, particularly with large sets of data, as each operation does not have to wait for a previous operation to complete. Higher-order n-bit logic operations through this directed logic architecture enable more complex algorithms to be created in a smaller space, where the PCM material reconfigures the operation, the FCD sets the data states, and the optical signal carries the result of the operation through to the output.

## Data Availability

All data generated from simulations are included in this published article
